# Evaluating the integration of palliative care in national health systems: an indicator rating process with EAPC task force members to measure advanced palliative care development

**DOI:** 10.1186/s12904-021-00728-z

**Published:** 2021-02-24

**Authors:** Natalia Arias-Casais, Eduardo Garralda, Miguel Antonio Sánchez-Cárdenas, John Y. Rhee, Carlos Centeno

**Affiliations:** 1 ATLANTES Research Program, Institute for Culture and Society, University of Navarra, 31080 Pamplona, Spain; 2 IdiSNA (Institute of Health Research of Navarra), Pamplona, Spain; 3 Department of Neurology, Massachusetts General Hospital and Brigham and Women’s Hospital, Harvard Medical School, Boston, MA USA

**Keywords:** Palliative care, Integration, Health systems

## Abstract

**Background:**

Palliative care (PC) development cannot only be assessed from a specialized provision perspective. Recently, PC integration into other health systems has been identified as a component of specialized development. Yet, there is a lack of indicators to assess PC integration for pediatrics, long-term care facilities, primary care, volunteering and cardiology.

**Aim:**

To identify and design indicators capable of exploring national-level integration of PC into the areas mentioned above.

**Methods:**

A process composed of a desk literature review, consultation and semi-structured interviews with EAPC task force members and a rating process was performed to create a list of indicators for the assessment of PC integration into pediatrics, long-term care facilities, primary care, cardiology, and volunteering. The new indicators were mapped onto the four domains of the WHO Public Health Strategy.

**Results:**

The literature review identified experts with whom 11 semi-structured interviews were conducted. A total of 34 new indicators were identified for national-level monitoring of palliative care integration. Ten were for pediatrics, five for primary care, six for long-term care facilities, seven for volunteering, and six for cardiology. All indicators mapped onto the WHO domains of policy and education while only pediatrics had an indicator that mapped onto the domain of services. No indicators mapped onto the domain of use of medicines.

**Conclusion:**

Meaningful contributions are being made in Europe towards the integration of PC into the explored fields. These efforts should be assessed in future regional mapping studies using indicators to deliver a more complete picture of PC development.

## Background

Palliative care (PC) development from a national-level perspective has been traditionally conducted using the domains proposed by the WHO Public Health Strategy for PC [1]. Therefore, national, regional, and global studies have utilized various indicators to assess PC development by mapping indicators onto the WHO domains of policies, education, use of medicines, and service provision [2]. These domains have been reported to correspond to the specialized development of PC [3]. However, recent discussions [2, 4] indicate that specialized development of PC may reflect a small part of the greater PC need, and comprehensive access to PC and its coverage may be more accurately measured by determining the level of integration of PC into national health systems.

The latest edition of the World Map assessing PC development globally used a classification system that included the concept of PC integration in terms of generalized provision [5]. The study used indicators reflecting various degrees of capacity building in palliative care to estimate integration of PC into national health systems [1]. The study concluded that 21 of 198 (11%) countries are in the preliminary level of integration of PC into mainstream provision, and 30 countries (15%) are in the level of advanced integration. However, integration was evaluated with regards to advances made in specialized service provision, access to medicine, education and policy and did not include the evaluation of PC integration into other fields and levels of the health systems.

Questions have been raised regarding whether specialized PC services have the capacity to provide PC for all those in need of PC [6]. In light of an increasing burden of serious health-related suffering [7], which is projected to double by the year 2060 to 48 million people every year (47% of all deaths globally) [8], there is a global call to health systems to develop strategies aimed at increasing access to PC at all levels. Strengthening health systems to integrate PC and expand coverage is particularly relevant in low-to-middle-income countries where, by year 2060, 87% of the global deaths are expected to take place [8].

Despite the absence of a consensus on the definition of PC integration, this concept has already permeated academic publications, international strategies, and advocacy groups. PC integration, seen as the accumulation of evidence suggesting that a person might receive relief from suffering due to advanced disease, at different levels of the system, through general or specialized services, may be examined using indicators which look at different areas and elements of the health system in relation to PC. The WHO included PC in the 2019 Astana Declaration on Primary Care [9], recognizing the importance of integrating PC into primary health care and increasing its access through wider coverage. The International Children’s Palliative Care Network has strived for the integration of PC into pediatrics [10]. The regional project Palliative Care for Older People in long-term care facilities (LTCF) and nursing homes in Europe explored the integration of PC into those facilities [11] and international organizations like the International Association for Hospice and Palliative Care has delivered advocacy statements at global health events like the World Health Assembly inviting Member States to integrate PC into primary care level [12].

Despite the increasing attention the topic of PC integration is gaining, to date, no study has addressed how to assess integration. The European Atlas of Palliative Care 2019 explored, for the first time, the integration of PC into other fields of the health system in Europe by focusing on pediatrics, long-term care facilities, primary care, cardiology, and volunteering. These fields were targeted due to their importance on providing PC in diverse care levels and for different types of patients (i.e. children, older adults at LTCF, and people in need of PC at the primary care level) [13, 14]. Furthermore, these areas were selected based on the existence of dedicated task forces in the EAPC, which informed the process and identified needs in the region.

National-level indicators to track progress or report on the status of current development in these fields are missing [2]. This paper aims to identify and design indicators capable of exploring national-level integration of PC into the areas mentioned above.

## Methods

Semi-structured interviews with EAPC task force members from pediatrics, long-term care facilities, primary care, cardiology, and volunteering were conducted. Next, a desk literature review to identify further indicators was conducted. A list of indicators was then given to EAPC taskforce members for rating.

### Semi-structured interviews with EAPC task force members

A process of consultation and semi-structured interviews with EAPC task forces leaders and members (in the case of cardiology) was designed. The EAPC Task Forces are working groups commissioned by the EAPC to develop research and discussion on specific topics based on their members’ interest and publications. Eleven task forces representatives were contacted and invited to participate in a semi-structured interview process regarding their respective expertise in the field (Table 1). Each EAPC Task Force usually has two leading experts, and both were included in the study by components of PC integration. In the case of Cardiology, two members of the task force were approached based on their interest in participating in the study. All experts were asked the following four questions: 1) How do you, as an expert, understand the concept of PC integration? 2) What are the most relevant indicators to assess PC integration within your respective areas of expertise? 3) Which aspects should be strengthened to allow integration of PC into your areas of expertise? and 4) What relevant articles could you recommend, including indicators to assess national-level integration or development within your field of expertise? The semi-structured interviews were recorded and transcribed. Two researchers (NA, EG) reviewed the recordings and transcripts to identify the indicators suggested by the experts.

**Table 1:** Experts participating in the identification and design process

Field	Experts
Pediatrics	Prof. Julia Downing Ms. Joan Martson Ms. Lizzie Chambers
Primary Care	Prof. Dr. Scott Murray Dr. Sebastiene Moine
Long-term care facilities	Prof. Katherine Froggatt Dr. Lieve van den Block
Volunteering	Ms. Leena Pelttari Ms. Ros Scott
Cardiology	Dr. Pablo Díez Villanueva Dr. Manuel Martinez Sellés

### Desk literature review

Additional indicators were extracted from peer-reviewed articles retrieved from a search in PubMed using the following search terms: Field of interest AND Europe AND Development AND/OR Integration [15]. Additionally, a “snow-ball” approach was used to gather articles not included in the original search that were suggested by the interviewed experts. Studies resulting from this hand-desk literature review and experts’ suggestions were assessed by two researchers (NAC, EG), and full-texts were used for the screening. Indicators were extracted from the studies and those indicators were included in a matrix chart.

### Rating process of identified indicators

A matrix of indicators extracted from interviews and the literature was organized and clustered by similarity. Repeated indicators were merged. The wording of the indicator reported in the literature was preserved and used over the one extracted from the interviews. A preliminary list including extracted indicators was sent to experts for proofreading and feedback. Experts’ suggestions were used to refine the wording used on naming the indicators, improving the questions proposed for their assessment, and adding additional indicators. An additional researcher from the team (CC) reviewed the list for consistency and clarity.

The identified indicators per area of competence were included into a survey sent to each EAPC task force member, who were then asked to rate (scale 1–9) each indicator using three variables: measurability, feasibility, and relevance. Relevance was defined as the degree to which the indicator was related to development on the area evaluated at a national-level. Measurability was defined as the degree to which the indicator could be quantified or measured. And feasibility was defined as the degree with which the indicator was easily obtained and collected by experts from the Atlas network.

Ratings per variable were used to calculate an average score called the Global Score (GS). Indicators scoring GS ≥ 7 (upper tercile) were selected, and indicators falling below the threshold were discarded. A preliminary list with selected indictors was sent back to EAPC task force members including the GS per indicator. Feedback on the results was requested and used to refine wording and further clarify the indicators.

During the 2018 EAPC Research Congress in Bern, a face-to-face rating was conducted using the updated list. The research group and the EAPC Task force members engaged in a discussion regarding the indicators. The EAPC task force members were asked to rate each indicator per GS. Indicators scoring GS ≥ 7 were selected.

## Results

A total of 11 experts participated in the semi-structured interviews and the rating of indicators (Table 1).

In total, 34 indicators were identified for the monitoring of palliative care integration into other fields of the explored areas. Ten were for pediatrics, five for primary care, six for long-term care facilities, seven for volunteering, and six for cardiology (Table 2).

**Table 2:** Identified indicators for the assessment of palliative care integration into health systems and rating

Field	Indicator	Global Score
**Pediatrics**	Vitality of pediatric palliative care associations	9.4
	Inclusion of PPC components in pediatrics curricula of specialization for doctors and nurses	9.2
	Existence of a PPC representative at the national PC association and vice versa	9.2
	Number and type of pediatric palliative care (PPC) services	9.1
	Availability of PPC training for neonatologists	9.1
	Existence of at least one national PPC association	9.1
	Number of specialized PPC consultants	8.8
	Existence of national standards and norms for the provision of PPC	8.7
	Existence of perinatal PC reference centers	8
	Existence of policies regulating pediatrics palliative care provision (P)	7.7
**Primary care**	Percentage of PC patients identified at the primary care level	8
	Time before death receiving PC at the primary care level	7.9
	Teaching of primary care PC components in the GP’s resident curricula	7.8
	Existence of incentives to promote early identification of PC patients at the primary care level (P)	7.4
	Teaching of primary care PC components to medical students	7.3
**Long-term care facilities**	Existence of official documents regulating PC provision in LTCF (P)	9.1
	Collaboration frequency between PC teams and LTCFs (estimate)	9
	Participation in international research projects assessing PC provision at LTCFs	8
	Existence of publications regarding PC provision at LTCFs	7.9
	Existence of PC training programs for staff working at LTCF	7.8
	Fund allocation for the provision of PC at LTCFs	7
**Volunteering**	Existence of training programs for PC volunteers	9.7
	Existence of a volunteers representative at the national PC association	9.6
	Number of PC volunteers	8.8
	Existence of data collection systems to track volunteers activity	8.7
	Number of volunteer hospices	8.6
	Availability of government funding to cover volunteering activities (P)	8
	Existence of compassionate communities	7.6
**Cardiology**	Existence of pioneering cardiology services providing PC	10
	Number of PC topics in national cardiology congresses and vice-versa.	10
	Number of publications regarding PC provision in cardiology services	8.6
	Existence of specific PC protocols for cardiology services (P)	7.6
	Frequency of collaboration between PC and cardiology services	7
	Existence of periodical meetings between the national PC and cardiology association	7

### Pediatrics

A total of ten indicators were identified, GS ranged from 7.7 to 9.4 and a median of 8.8 within the group. The highest scores within this domain were related to specific education or training regarding pediatric palliative care (PPC), engagement and activity of national associations, and service provision. The highest rated indicator was the vitality of pediatric PC associations (GS = 9.4). Vitality has been defined in the scientific literature as the amount of professional activity or engagement that the national PC association has on promoting PC at the local level for example through scientific congresses, educational and informative activities [16]. The inclusion of pediatric PC components on the specialization curricula of future pediatric nurses and doctors scored a high rating from among the indicators (GS = 9.2) as well as the existence of a representative of PPC at the national PC association (GS = 9.2). The number and type of pediatric palliative care services and the availability of PC training for neonatologists received high scores as well as the existence of at least one specific pediatric palliative care association.

### Primary care

Five indicators were identified with GSs ranging from 7.3 to 8 and a median GS of 7.6. The highest rated indicators within primary care were those related to primary care preparedness in identifying, in a timely fashion, patients in need of PC. The highest rated indicator was the percentage of PC patients identified at this level (GS = 8) followed by how much time before death a patient receives PC at the primary care level (GS = 7.9). The following indicator on the list was on teaching of primary care PC components in the GP’s resident curricula (GS = 7.8) and the last was on the mechanisms in place to promote the early identification of patients at the primary care level (GS = 7.4).

### Long-term care facilities (LTCF)

Six indicators were identified, with GSs ranging from 7 to 9.1 and a median GS of 8.1. For the assessment of the integration of PC into long-term care facilities, the highest rated indicator was the existence of legal frameworks to regulate the provision of PC at long-term care facilities (GS = 9.1). The collaboration among these types of facilities and PC teams (GS = 9) and the existence of training programs for the staff at these institutions (GS = 7.8) also scored high ratings. In this area of competence, the existence of international peer-reviewed research on the topic within countries was suggested as an important indicator to assess the integration (GS = 7.9).

### Volunteering

A total of seven indicators were identified, with GSs ranging from 7.6 to 9.7 and a median GS of 8.7. The existence of training programs or curricula for PC volunteers was rated with the highest score (GS = 9.7) on this area of competence followed by the existence of a representative for volunteers at the national PC association (GS = 9.6). The number of volunteers in the country was also selected as an indicator to assess the integration of this field into PC (GS = 8.8), and the existence of data collection systems to track the number of volunteers and activities was also highly rated (GS = 8.7).

### Cardiology

A total of six indicators were identified, with GSs ranging from 7 to 10 and a median GS of 8.3. For this area of competence, the existence of a pioneering cardiology service that provides PC was rated as the highest national-level indicator on the integration (GS = 10). Also, highly scored were the participation of PC topics in cardiology congresses (GS = 10) and the number of scientific peer-reviewed publications on the provision of PC through cardiology services (GS = 8.6).

## Discussion

Current discussion points out that PC development does not only focus on the advances made at the generalized provision with regards to the domains suggested by the WHO. Generalized development also refers to the basic foundation of policies, use of medicines, education and service provision to allow for a comprehensive, yet basic, coverage. However, after general provision is achieved, health systems should allow for integration into other fields of the health system to guarantee that all those in need can access it. This study shows some fields in which PC has already started to be integrated in Europe and proposes a list of indicators to assess its integration in national-level assessment studies.

Some studies have previously aimed and assessed PC integration into other levels of health systems (LTCF, PC) [17, 18]. The WHO has also strived to promote integration and drafted three manuals for the integration of PC into pediatrics, primary care, and emergency settings [19, 20]. More recently, the World Map delivered a categorization of countries into PC development levels that included categories for preliminary and advance integration [5]. These categories were defined based on the implementation of the following indicators: a) ratio of services per population as recommended by international organizations, assuring wide geographically spread; b) existence of a national palliative care strategy that is implemented, updated, and evaluated; c) a wide availability and consumption of all types of opioids; d) the existence of a recognized medical specialty in PC and the inclusion of PC education at the undergraduate level in the medicine and nursing curricula [21]. These indicators correspond to domains of the WHO Public Health strategy for PC: policy, education, specialized service provision, and use of medicines. We argue that the concept of integration should also be measured through generalized PC provision as assessed through each of the WHO domains as well as a specific evaluation that depicts the way and the level in which PC permeates other areas of the health systems. Such indicators should be included in a comprehensive framework for the monitoring and evaluation of PC development and included in global strategies [7]. Other strategies for global health priorities on the fight against chronic conditions, such as HIV/AIDS and cancer, can shed light on the kind of indicators to aim at in the future for the assessment of PC integration into health systems.

This study explored important areas where PC is being provided and integrated. The evaluation of the current national-level status of the integration into the areas explored shows meaningful contributions made on the coverage of patients in need. Relevant examples are the provision in pediatrics, the relevance of volunteers at the community level as well as the role of long-term care facilities on covering the needs of their residents.

These indicators are the first to be proposed for national-level monitoring of the integration of PC in specialized fields outside of generalized PC provision and are presented as a baseline from which to start a discussion. The scope of this study included the identification and formulation of these indicators rather than the assessment of their quality. Further research should address this issue. Additionally, only five areas of competence were selected for this study based on the availability of experts who were part of the EAPC task forces. However, future studies should address other areas, for instance, geriatrics and neurology. Additional research should be targeting at improving the indicators’ definitions. For instance, future research can aim at finding a consensus on the denominator or benchmark for the percentage of PC patients identified at the primary care level. Improving definitions can set a blueprint for the monitoring and evaluation of PC integration in different contexts.

Nevertheless, the identified indicators show relevant areas to address the assessment of PC integration. Some of them resemble the WHO domains already used for generalized development, mainly education and policy. The policy component is present in all areas of competence and takes the shape of the existence of legal frameworks for the provision of PC (i.e. pediatrics and long-term care facilities), the existence of incentives or protocols (i.e cardiology and primary care), and the availability of funding to support provision (i.e. volunteering). Similarly, the component of education is present in all areas of competence. In the case of primary care, and pediatrics the focus is on the inclusion of PC components on the training curricula of the health care work force for future pediatricians and doctors. Furthermore, education remains present also in long-term care facilities, volunteering, and pediatrics where PC training for volunteers and long-term staff was shown to be relevant for the provision of PC. In the case of pediatrics, specific training for neonatologists was rated as an important indicator. This shows the importance of integrating this sub-specialization into the provision of PC in pre-natal and newborn health to address the suffering of patients and families dealing with fatal diagnosis in the early stages of life.

The only field of competence with a specific indicator relating to the WHO domain of services was reported within pediatrics. No indicators were associated with the WHO’s use of medicines domain. In light of the overlapping domains, future research should focus on identifying more indicators for the assessment of PC integration into other fields of the health systems and attempt to suggest a classification or framework that could potentially be used to explore other areas of competence.

## Conclusion

Measuring the development of palliative care through indicators has increasingly received attention in recent years, and it is necessary to assess the level of integration of palliative care into the national health systems. This study identified 34 new indicators to assess pediatric palliative care, primary palliative care, long-term care provision, volunteering, and the integration of palliative care into the cardiology field.

## Data Availability

Data management and sharing All EAPC Atlases data can be accessed from http://dadun.unav. edu/handle/10171/56787 or can be asked from egarralda@ unav.es.
